# Compulsory health insurance in the Central and Eastern European countries: the similarity and the difference with Social Health Insurance

**DOI:** 10.3389/fpubh.2026.1761310

**Published:** 2026-03-16

**Authors:** Sergey Shishkin

**Affiliations:** National Research University Higher School of Economics, Moscow, Russia

**Keywords:** Bismarck model, compulsory health insurance, government health financing scheme, health financing regulation, Social Health Insurance

## Abstract

**Background:**

According to the conventional point of view, compulsory health insurance (CHI) models in the Central and Eastern European (CEE) countries are classified as Social Health Insurance (SHI) schemes. However, the absolute dominance of the state in their regulation in many CEE countries calls into question such a qualification. The existing approaches to the definition of SHI are insufficient to clearly distinguish existing CHI models from government health financing schemes as well as to identify their exact similarities and differences with SHI models in Western Europe (WE).

**Methods:**

The new conceptual framework is used to compare the similarities and differences between the SHI models in CEE and WE countries. The characteristics of financial flows regulation are considered to distinguish SHI from government healthcare financing schemes. Different CHI models are compared by using three different types of regulation (state, societal, market) of the three main functions of health financing system (collection, pooling, purchasing), and by types of financing agents. A qualitative, cross-country analysis is implemented with use the data from the WHO Health system reviews Health Systems in Transition.

**Results:**

In almost all countries considered, state regulation is dominant in the implementation of collection and pooling functions. A variety of combinations of state, societal, and market regulation is observed in the purchasing of medical services. State regulation dominates in most CEE countries, societal regulation dominates in some of them and in almost all WE countries. In most CEE countries and in some WE countries state regulation of purchasing is complemented by market regulation. Based on these characteristics, four types of SHI models are identified (State, Etatist, Bismarckian, and Quasi-Market).

**Conclusion:**

The dominance of state regulation in the CHI models in almost all CEE countries is not a sufficient reason to qualify these models as hybrid financing systems. They retain fundamental differences from government financing schemes: the systems of financial flows are separated from state budgets and operate according to the rules set specifically for these systems.

## 1 Introduction

In most Central and Eastern European (CEE) countries, after the collapse of socialism, compulsory health insurance (CHI) was introduced to substitute or supplement government health financing systems (1). In the latter, the financial sources are tax revenues; the financial flows from state budgets to pay medical service providers are administered by government bodies. According to the conventional point of view, CHI models in CEE countries are classified as Social Health Insurance (SHI) systems. In the terms currently used by the OECD and WHO in national health accounting, they are variants of the same type of health financing scheme—the SHI scheme (2). This scheme is based on the Bismarck model of SHI. It includes the compulsory participation of certain categories of citizens, compulsory income-related insurance contributions, which are paid by employers and employees, and financing medical care for insured persons from these funds. The insurers in this model are non-governmental insurance funds. SHI is managed by self-governing corporate bodies with equal participation by representatives of employers, employees, and providers of medical services (3).

The actual SHI models in European countries differ from the original Bismarck model (4, 5). Insurance contributions of employers and employees are supplemented by budget transfers which make up from 25 to 50% or more of the income of SHI systems in Europe (6). SHI systems are increasingly dependent on general taxation as source of their financing (7). The government sets the rate of insurance premiums, the rules for their redistribution between insurers (risk adjustment), and regulates the interaction of insurers and providers. In CEE, the design of the CHI models differs significantly from the design of both the original Bismarck model and from the current SHI models in Western Europe (WE). In almost all CEE countries, CHI funds were initially created as public organizations. In Bulgaria, Hungary, and Slovenia, one national CHI fund was created initially (8–10). In Estonia, Lithuania, Poland, and Romania, the initially created central and territorial CHI funds were then combined into a single national CHI fund, accountable to the government (11–14). In Bulgaria, Estonia, Hungary, Lithuania, Poland, and Russia, the national CHI funds, accountable to the government, have, over time, been fully integrated into the government health care administration: they were subordinated to the respective MoH or became a department thereof (9–13, 15). In CEE, government bodies play the key role in regulating CHI and in its operational management. This gives grounds for the emergence of assessments of CHI models in CEE countries as little different from the government health financing systems. For example, in Russia some experts classify the existing СHI model as a budget-insurance hybrid (16), an intermediate model (17), and even as a de facto budgetary one (18). Such assessments were caused by the fact that in Russia an attempt was made to introduce a competitive CHI model, but it has evolved substantially with the dominant trend of increasing state regulation inherent in the government health financing system and a decreasing role of characteristics of the market-driven competitive model (19).

The absolute dominance of the state in the regulation of CHI models in many CEE countries raises questions about their qualification. Is it justified to consider them as a type of SHI? Or they are hybrid systems that combine SHI institutions and institutions of government health financing systems? Or do they represent a special health financing system that is distinct from both SHI and government health financing system?

In the literature, there is a distinction between four types or models of health care systems: Beveridge, Bismarck, National Health Insurance, and Private Health Insurance (20–22). The National Health Insurance model is considered to contain elements of both the Beveridge and Bismarck models. However, it should be emphasized that this typology applies specifically to health care systems. Thus, the National Health Insurance is defined as a health care system in which payment comes from a government-run insurance program, and medical care is provided by private-sector providers. It should also be noted that some publications question the usefulness of distinguishing between the Bismarck and Beveridge systems in modern conditions (23) and even directly assert that the conventional distinction between government health financing systems and SHI is no longer meaningful (6).

The qualification of CHI models in CEE countries is not a purely theoretical question. In some CEE countries, particularly in Russia, the question of whether the health financing system is classified as an insurance or government system has acquired real political significance. The qualification of a CHI model as hybrid or non-insurance model serves as the basis for a negative assessment of the CHI model and for proposals for its dismantling and replacement with a government financing model.

The economic shock caused by COVID-19 raised the question of the need to broaden the public revenue base for healthcare systems (6). The peculiarities of economic development in many countries may require significant changes in the sources of health financing in favor of general taxation, new earmarked taxes, etc., and corresponding changes in previously created health financing models. This actualizes the question of the differences and typologies of health financing systems.

## 2 Theoretical background and literature review

Traditionally, the source of funds has been considered the main difference between SHI and government health financing systems; general taxation for government systems and earmarked income-related contributions paid by employers and employees for SHI. The traditional approach postulates that the source of funding correlates with how these systems are organized (24).

The theory of SHI has expanded the grounds for distinguishing between SHI and government health financing systems. The main characteristic of SHI is interpreted as the presence of societal institutions of governance and societal values: the management of SHI is carried out by quasi-public bodies formed on the principles of social partnership (representing the interests of employers and employees), which are based on social values (self-regulation, pluralism, co-participation, etc.) (25, 26). When describing the organization of SHI systems, in addition to the source of financing, mandatory membership for certain categories of people was highlighted, as well as the public nature of setting the rate of contribution, a ban on collection contributions depending on individual risk of morbidity, and the separation of insurers from the government budget system (27). These characteristics and the identification of three main types of health financing systems (government, SHI, and private) were sufficient to classify country models of health financing up to the end of the 1980s.

However, the development of SHI models in WE and the transition from government health financing systems to SHI in CEE countries meant that health financing models emerged that differed from the original Bismarck SHI model. This required the creation of a new conceptual framework for analyzing health financing systems.

Different approaches have been proposed to characterize modern health financing systems, and SHI in particular. Gerlinger and Schmucker (4) define the core characteristics of SHI not as absolute, but as relative. The core characteristics of SHI are defined in terms of dominance: the dominant role of insurance premiums as a source of SHI funds and the dominant role of societal regulation in managing the SHI system. But the notion of dominance is not clearly defined and a measure of dominance is not set. With this approach, the criteria for identifying different types of healthcare financing systems become blurred.

The health accounts methodology proposed by the OECD and WHO expands the set of the criteria used for the typology of health financing schemes (2). The following criteria were proposed for distinguishing financing schemes: (1) mode of participation, (2) benefit entitlement, (3) basic method for fund-raising, (4) mechanism and extent of pooling funds. The typology of health financing schemes includes: (1) government schemes, (2) Social Health Insurance, (3) compulsory private insurance, (4) compulsory medical saving accounts, (5) voluntary health insurance schemes, etc. In addition to health financing schemes, the characteristics of the institutional structure (the types of financing agents, etc.) are used to describe national health financing systems. However, they are not used as the criteria for a typology of health financing schemes.

The characteristics of CHI models in most CEE countries, according to the above criteria, are very close to the characteristics of government health financing schemes. Thus, the difference between the mandatory mode of participation in CHI models with complete or almost complete coverage and the automatic mode of participation in government schemes is formal. Similarly, the distinction between non-contributory and contributory benefit entitlement is also formal. The difference in sources of financing remains, but since the budget of CHI systems in CEE countries is planned by the government, and a significant part of the CHI system funding comes from state budgets, the collection of funds in CHI, differing in methods, does not functionally differ from government schemes. In both cases, the total amount of funds is determined as part of the overall budget process.

In many publications the health financing systems are distinguished as part of the larger task of finding new foundations for classifying health care systems (28–30). A recent review identified 42 typologies of health care systems (31). Among the publications on this topic, the works of Rothgang et al. (32) are very often cited. They proposed using three dimensions to characterize health care systems: (1) financing, (2) provision of services, (3) regulation, and three types of actors: (1) state, (2) societal, (3) private (33). When considering health care systems, the dominant actor is fixed in each for the three highlighted dimensions. Different health care systems are characterized by different combinations of these characteristics. The authors identify five types of health care systems: National Health Service, National Health Insurance, Social Health Insurance, Etatist Social Health Insurance, and Private Health Care System. Examples of countries with Social Health Insurance systems are Austria, Germany, and Switzerland. Examples of countries with Etatist Social Health Insurance are Belgium, Czechia, Estonia, France, Hungary, Netherlands, Poland, Slovakia. With this approach, the CHI systems in CEE countries are again in the same group as the SHI models in the WE countries, and the question of the grounds for such an association remains open.

Kutzin proposed a new theoretical framework for considering health financing systems based on the functional analysis paradigm (34). It is used in many research papers and in official WHO publications. According to this approach, any health financing system performs the following functions: collection of funds, pooling of funds, purchasing of services, and provision of services. The differences between the health financing systems are analyzed as differences in the mechanisms used to perform these functions. This approach has been constructive for describing and comparing changes in health care financing systems in different countries. However, the differences between the types of such systems dissolve in the differences between alternative mechanisms for implementing universal functions.

To analyze the similarity and difference between CHI models, further structuring of the theoretical and methodological framework for their consideration is required. An analysis of the approaches to identifying common characteristics of modern CHI models shows that, despite the obvious differences between these approaches, they contain a common methodological component: specific regulatory characteristics. This is the setting of a specific source of financing for SHI, a specific basic method for fund-raising, a specific mode of participation and benefit entitlement, the use of a specific type of regulation. Therefore, it seems productive to look for more informative differences between health financing systems for a more detailed structuring of the regulation the financial flows in these systems.

## 3 Methods

### 3.1 Terms

The term “compulsory health insurance model” is used here to describe the characteristics of the CHI in a particular country. The terms “health financing system” and “health financing scheme” are used synonymously.

### 3.2 Subject of research

In this article the following countries as Central and Eastern European countries with CHI are considered: Bulgaria, Czechia, Croatia, Estonia, Greece, Hungary, Lithuania, Moldova, North Macedonia, Poland, Russia, Romania, Serbia, Slovakia, Slovenia. To answer the central question of the study about the similarity and the difference in CHI in these countries with Social Health Insurance, Western European (WE) countries with Social Health Insurance systems are also included in the consideration: Austria, Belgium, France, Germany, Luxembourg, the Netherlands, and Switzerland.

### 3.3 Data sources

Publications in the WHO series “Health system review. Health Systems in Transition” (HIT) (8–15, 35–49) serve as sources of information about CHI models. The specific characteristics of the CHI model for each country are based on the description of the health care system in that country which is presented in the corresponding HIT. These publications contain a structured overview of the statutory health financing system including revenue collection, pooling and the allocation of funds among pools/purchasers, purchaser–provider relations, the main actors in the health system, and their role in setting the rules of collection, pooling, and purchasing (50).

The characteristics of a country’s CHI model are reviewed as of the year of the latest HIT release. All European countries with CHI are included in the analysis, with the exception of Albania, Bosnia and Herzegovina, and Montenegro, for which HIT is released earlier than 2010 or is missing.

### 3.4 Methodology

A new approach is used for a comparative analysis of CHI models by integrating elements of several approaches proposed in the literature to characterize differences between health financing systems: functional analysis paradigm (34), analysis of the types of health care regulation (32, 33), and the types of financing agents (2). To characterize the differences between health financing systems, three main functions are considered: collection, pooling, and purchasing and three types of regulation are analyzed: state, societal, and market. State regulation includes decisions made by government agencies using their authority (hierarchical decisions). Societal regulation is collective decisions made through collective negotiations between participants of the health financing system (representatives of employers and employees, government bodies, purchasers and providers of medical services, etc.). Market regulation includes decisions made through individual agreements between participants of the health financing system.

For each country-specific CHI model, the types of regulation are identified which are used in relation to making the following decisions.

Collection:

- Deciding on coverage (setting the benefit package: the content and range of medical care offered to patients);
- Setting contribution rates for CHI.

Pooling:

- Setting the rules for allocation of funds.

Purchasing:

- Setting the lists of reimbursable services and medicines;
- Setting the prices/tariffs;
- Selecting the providers;
- Determining the volumes of services.

The types of regulation used to make these decisions are identified based on an analysis who sets these rules. If this is done by government agencies, then obviously there is state regulation. If the decisions are made by corporate bodies or as a result of collective bargaining between government agencies and corporate bodies, then they are classified as societal regulation. If decisions are made by agreement between two parties, for example, an insurer and a medical care provider, who have some options for choosing the parameters of this agreement, then these decisions are qualified as market regulation. If, when making the decisions outlined above and establishing the corresponding rules, different decisions or different components of these rules are subject to different types of regulation, then all types of regulation used are indicated.

For each function of the healthcare financing system, the dominant type of regulation is identified by a qualitative assessment of the contribution of each type of regulation to setting rules used for implementation this function. In some cases, when the data contained in HIT does not allow this, the dominant type of regulation is not highlighted.

To distinguish CHI models, the following types of financing agents are considered:

- Government unit (subordinated to or integrated into a MoH);
- СHI fund accountable to government;
- CHI fund with independent status (quasi-public or private nonprofit);
- Health insurance company.

As a result of this analysis, each country model of the CHI is described by a certain combination of the selected characteristics. Next, a typology of the country’s CHI models is proposed based on the similarity of the combinations of types of regulation used and types of financing agents.

## 4 Results

### 4.1 Collection

In all the CEE countries, state regulation is dominant in the implementation of collection function (Table 1). The benefits package is set by the government. The rates of CHI contribution are set by law. Societal regulation is used to perform the collection function only in two CEE countries. In North Macedonia, the detailed scope of care covered by CHI is defined through a trilateral committee including representatives of the MoH, the Health Insurance Fund and the Doctor’s Chamber. In Slovenia, the list of health care services involving cost-sharing through co-payments is accepted by the Health Insurance Institute of Slovenia Assembly, comprising representatives of employers, the insured population, etc.

**Table 1:** The results of the structural analysis of CHI models in Europe.

Country	Healthcare financing system functions									Financing agent(s)			
	Collection			Pooling			Purchasing			Government unit	CHI fund accountable to government	CHI fund with independent status	Health insurance company
	Type of regulation			Type of regulation			Type of regulation						
	State	Societal	Market	State	Societal	Market	State	Societal	Market				
Group 1													
Greece	**+**			**+**			**+**		+	+			
Hungary	**+**			**+**			**+**			+			
Lithuania	**+**			**+**			**+**			+			
Poland	**+**			**+**			**+**		+	+			
Republic of Moldova	**+**			**+**			**+**		+		+		
Romania	**+**			**+**			**+**		+		+		
Serbia	**+**			**+**			**+**		+		+		
Slovakia	**+**			**+**			+		**+**				+
Group 2													
Bulgaria	**+**			**+**				**+**	+	+			
Croatia	**+**			**+**			+	+	+		+		
Estonia	**+**				**+**		**+**	+	+	+			
North Macedonia	**+**	+		**+**			+	+	+		+		
Russia	**+**			**+**			**+**	+	+	+			+
Slovenia	**+**	+			**+**			+	**+**		+		
Group 3													
Austria	**+**			**+**				**+**				+	
Belgium	**+**			**+**	+		+	**+**	+			+	
Czechia	**+**			**+**				**+**	+			+	
France	**+**			**+**				**+**				+	
Germany	**+**	+	+	**+**				**+**	+			+	
Luxembourg	+	**+**		**+**			+	**+**				+	
Group 4													
Netherlands	**+**		+	**+**					**+**			+	+
Switzerland	+		**+**		**+**		+	**+**	+				+

The dominant type of regulation of the corresponding function of the healthcare financing system is highlighted in bold.

Source: generated by the author based on data from (8–15, 35–49).

In WE countries considered, with the exception of Luxembourg, state regulation is also dominant in the implementation of collection function. Societal regulation is still used in only two WE countries. In Germany, state regulation of benefits package and rates of CHI contribution is complemented by societal and market regulation. German health insurers (sickness funds) may charge their clients a supplementary contribution if the expenditures exceed the allocations through the central reallocation pool. Since sickness funds are the nonprofit corporate organizations the setting of supplementary contributions is an act of societal regulation. Sickness funds have the right to offer a range of rate options. Insured persons can opt for an individual tariff, and this choice serves as a means of competition between sickness funds (market regulation). In Luxembourg, as in the original Bismarck model, societal regulation dominates: the contribution rate is set by the SHI Council of Administration, which consists of elected representatives of employers, insured employees, and self-employed persons in equal shares, and is headed by a government representative. Market regulation in addition to the state one is used in the Netherlands and Switzerland. In the Netherlands, the income-related employer and employee contributions are set by the law (state regulation). The health insurers offer various health plans and set additional insurance premiums for them (market regulation). In Switzerland health insurers determine insurance premiums (market regulation), approved by a government agency (state regulation).

The dominance of state regulation is common to both CEE and WE countries in the implementation of pooling function. The difference is that in most CEE countries other types of regulation are not used, while in four of the seven WE countries considered, state regulation is combined with societal and/or market one.

### 4.2 Pooling

The pooling function in most of CEE countries is subject to state regulation. The government sets the rules for allocation of CHI funds. In Estonia, and Slovenia, setting these rules is subject to societal regulation. In Estonia, fund allocation is decided by the Health Insurance Fund Supervisory Board, which includes tripartite representation of the state, employers, and organizations of insured individuals. In Slovenia, the fund allocation is decided by the Assembly of the Slovenian Institute of Health Insurance, consisting of representatives of employers and the insured population.

In almost all WE countries state regulation is dominant in the implementation of pooling function. Switzerland is an exception. The common pool of contributions is managed by the Common Institution representing the interests of health insurers (societal regulation).

The dominance of state regulation in the implementation of pooling function is similar for most CEE and WE countries.

### 4.3 Purchasing

A variety of combinations of state, societal, and market regulation can be observed in the purchasing of medical services. In most CEE countries, state regulation is the dominant type of purchasing regulation. In some CEE countries (Bulgaria, Czechia, North Macedonia), societal regulation dominates in purchasing. In Bulgaria, the National Health Insurance Fund is managed by the Supervisory Board which consists of representatives of employee, employer and patient organizations and the government. It negotiates with the professional associations of physicians and dentists and the national framework contracts with health care providers. In Czechia, a committee consisting of health insurance fund representatives, health care providers, and professional medical associations, makes recommendations to CHI insurers for choosing providers. The insurers negotiate with specific groups representing providers (hospitals, physicians and outpatient care specialists) for the conditions of reimbursement. In North Macedonia, the CHI Fund negotiates with professional chambers (medical, pharmaceutical, and dental) on the details of contracts with providers.

Societal regulation of purchasing is also used in Croatia, Estonia, Russia, and Slovenia, but is not dominant. In Croatia, the CHI Fund, in cooperation with medical associations, determines the price list of reimbursed medical services, but final decisions are made with approval of the MoH. Market regulation of purchasing is also used: the CHI Fund contracts providers on a competitive base. The Estonian Health Insurance Fund negotiates standard contract terms with provider associations, but the list of providers, the list of reimbursed health care services, medicines, medical devices, and the medicine price ceilings are determined by the MoH and Labour. In Russia, contracts between health insurance companies and providers (market regulation) in each region are determined by a regional commission composed of representatives of the regional health authority, insurance companies, medical care providers, and medical associations (societal regulation). However, the commission’s decisions are determined by the regional health authority (ie, state regulation dominates) (19). In Slovenia, the MoH, the Health Insurance Fund, the association of providers, and medical and pharmaceutical chambers conclude a General Agreement which defines the total volume of services to be provided (societal regulation). The exact type and volume of services, the tariffs, and the methods of payment are set in contracts between the Health Insurance Fund and individual providers (market regulation).

In most CEE countries, societal regulation of purchasing is not used, but state regulation is complemented by market one. Insurers enter into contracts with individual suppliers, which determine the type, scope, and quality of provider activities. The possibilities of selective contracting providers and negotiating services with them occur within a narrow framework—on the basis of volume, quality, and price—set by government agencies or agreements between insurers and associations of providers, physicians, and patients. However, in Slovakia, market regulation of purchasing dominates. The MoH sets the minimum volumes of medical care and a list of selected public providers which must be contracted. The insurers are allowed to use their own payment mechanisms, to set their own prices, and to select other providers for provision medical care in excess of established minimum volumes.

In WE countries, with the exception of the Netherlands, societal regulation dominates in purchasing. Market regulation complements societal regulation of purchasing in Belgium, Germany, and Switzerland. In the Netherlands, the purchasing function is implemented only by market regulation instruments.

Thus, in the implementation of the purchasing function, the difference between CEE and WE countries is significant. In the former, almost all of them, state regulation dominates, while in the latter, almost all of them, societal regulation dominates.

### 4.4 Status of financing agent(s)

In most CEE countries health insurers are either public organizations, accountable to governments, but with a certain autonomy, or government units subordinated to or integrated into MoH. In the Russian CHI model, the functions of an insurer are divided between two types of actors: (a) federal and regional CHI funds, which are public institutions accountable to federal and regional governments respectively, and (b) health insurance companies, which are usually private for-profit organizations. In Slovakia, there are three insurers, which are joint stock companies, one of which is owned by the state. Czech health insurance funds are quasi-public, self-governing bodies.

By contrast, in most of WE countries, financing agents are CHI funds with independent status. Belgian health insurers are private, non-profit organizations. German health insurers are non-profit corporate institutions. The main health insurer in France is a public institution with financial autonomy, and the second one has a special status (mutualité sociale). In Luxembourg, insurers are public law bodies. In the Netherlands, insurers are non-profit cooperatives, in which insured persons are members of a board which controls the management. There are also the financing agents which are private for-profit organizations. In Switzerland, health insurers are private companies.

There is clear difference between CHI models in CEE and in WE countries by the status and by the corresponding type of governance of financing agents. In most CEE countries, the governance of financing agents is centralized. In all WE countries, and in Czechia and Slovakia the governance of financing agents is autonomous.

### 4.5 Distinguishing CHI country models into groups

Considering the structural characteristics of the CHI country models, four groups can be distinguished (Table 2). Three criteria are used to identify them:

- Composition of functions in which each type of regulation is used;
- Composition of functions in which each type of regulation dominates;
- Type of financing agents status and governance.

**Table 2:** The dominant types of regulation of healthcare financing system functions and the types of financing agent(s) governance in CHI country models.

Country	Dominant type of financing system functions regulation			Type of financing agent(s) governance
	Collection	Pooling	Purchasing	
Group 1				
Greece	State	State	State	Centralized
Hungary	State	State	State	Centralized
Lithuania	State	State	State	Centralized
Poland	State	State	State	Centralized
Republic of Moldova	State	State	State	Centralized
Romania	State	State	State	Centralized
Serbia	State	State	State	Centralized
Slovakia	State	State	Market	Centralized
Group 2				
Bulgaria	State	State	Societal	Centralized
Croatia	State	State	Mixed	Centralized
Estonia	State	Societal	State	Centralized
North Macedonia	State	State	Mixed	Centralized
Russia	State	State	State	Centralized
Slovenia	State	Societal	Market	Centralized
Group 3				
Austria	State	State	Societal	Autonomous
Belgium	State	State	Societal	Autonomous
Czechia	State	State	Societal	Autonomous
France	State	State	Societal	Autonomous
Germany	State	State	Societal	Autonomous
Luxembourg	Societal	State	Societal	Autonomous
Group 4				
Netherlands	State	State	Market	Autonomous
Switzerland	Market	Societal	Societal	Autonomous

Source: generated by the author based on data from (8–15, 35–49).

Group 1: state regulation dominates in the implementation of all three functions, societal regulation is not used, financing agent is government unit or CHI funds accountable to government, their governance is centralized. This group includes the CHI models in seven CEE countries: Greece, Hungary, Lithuania, Poland, Republic of Moldova, Romania, Serbia, Slovakia. The latter is somewhat different from the other countries in this group, the market regulation but not state one dominates in the implementation of purchasing function. But due to the lack of societal regulation, Slovakia can be included into this group.

Group 2: state regulation dominates in the implementation of two or all three functions, societal regulation is used in the implementation of at least one function, financing agents are government unit or CHI funds accountable to government, their governance is centralized. This group includes the CHI models in six CEE countries: Bulgaria, Croatia, Estonia, North Macedonia, Russia, Slovenia.

Group 3: state regulation dominates in implementation of pooling functions, societal regulation dominates in implementation of purchasing function, financing agents are CHI funds with independent status and autonomous governance. This group includes CHI models in five WE countries (Austria, Belgium, France, Germany, Luxembourg) and one in CEE country (Czechia).

Group 4: use of market regulation to implement the functions of collection and purchasing and its dominance in the implementation of one of this function, financing agents are CHI funds with independent status and/or health insurance companies. The Netherlands and Switzerland form this group.

## 5 Discussion

In all the countries considered, the functions of collection and pooling funds are regulated by the state, while other types of regulation of these two functions are applied only in eight countries (Estonia, North Macedonia, Slovenia, Belgium, Germany, Luxembourg, the Netherlands, Switzerland). In most CEE countries, state regulation is dominant in regulating purchasing, and CHI financial agents are accountable to the government or even integrated into the public health administration. However, in almost all WE countries, societal regulation is still dominant in implementing the purchasing function, and financial agents have the status of quasi-public or commercial organizations independent of the state.

Are these comparative characteristics sufficient grounds to qualify the relevant CHI models in CEE as hybrid systems that combine the institutions of the SHI and the government financing system? No. A comparison of all CHI models allows us to conclude that there is a difference between the CHI models in CEE countries and the government health financing system. Attention should be paid not only to the dominance of the state regulation of the collection, pooling, and purchasing functions, but to the subject of regulation itself. What is common to all the CHI models considered is the existence of specific rules for financial flows, which are different from the rules for implementing these functions within the government financing system.

In the literature, when characterizing SHI funds, there is reference not to their traditional source—employee and employer income-related contributions—but to funds earmarked for health (51). Hsiao and Shaw note “the free-standing nature of SHI financial operations” (51). Norman and Weber emphasize that the financial resources in the SHI are earmarked, pooled, and administered in a separate budget (52). In SHI, a fund is created separate from the state budget, which is managed by a specialized financing agent.

These characteristics mean that SHI has specified rules for financial flows:

- The specification of categories of insured persons participating in the financing system;
- The specification of the sources and amounts of mandatory contributions for each category of insured;
- The pooling of contributions separate from the state budget;
- Setting rules specifically for the allocation of these contributions.

The presence of such separate rules for the financial flows in the healthcare financing system unites the CHI models and distinguishes them from the government health financing system. This gives grounds for the assertion that the CHI models in CEE and in WE countries can be combined with the general concept of SHI.

The identification of four groups of CHI models provides grounds for distinguishing four different types of such models given the similarity of their structural characteristics (Table 3).

**Table 3:** Comparative characteristics distinguishing different SHI models.

Social Health Insurance models	Regulation of financing functions	Financing agent(s)	Countries
S-type (State) Social Health Insurance	Dominance of state regulation of implementing all functions. Lack of societal regulation	Government unit or CHI fund account-able to government	Greece, Hungary, Moldova, Lithuania, Poland, Romania, Serbia, Slovakia
E-type (Etatist) Social Health Insurance	Use all three types of regulation	Government unit or CHI fund account-able to government	Bulgaria, Croatia, Estonia, North Macedonia, Russia, Slovenia
B-type (Bismarckian) Social Health Insurance	Dominance of societal regulation in the implementation of a purchasing function	CHI fund with independent status	Austria, Belgium, Czechia, France, Germany, Luxembourg
Q-type (Quasi-Market) Social Health Insurance	Using market regulation to implement collection and pooling functions	Private companies	Netherlands, Switzerland

The first group is characterized by the state regulation of all three functions, by the absence of societal regulation, and by the fact that CHI funds are public organizations. In most countries of this group, CHI funds are integrated into the public health administration system. That is why this group can be designated as S-type (State) Social Health Insurance.

The CHI models in the seсond group use all three types of regulation and their CHI funds are public organizations. Therefore, this group of models can be designated as E-type (Etatist) Social Health Insurance. This term is borrowed from Böhm et al. (33), but it is used in a different theoretical and methodological framework.

In the third group, the highlighted characteristics are the dominance of societal regulation in the implementation of the purchasing function and the independent status of SHI Funds. The current CHI models in these countries differ from the original Bismarck model in a number of ways, but it is here that the CHI models are closest to the original prototype. Therefore, it is proposed to designate this group of country models as B-type (Bismarckian) Social Health Insurance.

The fourth group uses market regulation tools to implement the functions of collection and purchasing, and insurers in these models are private companies. The composition of this group coincides with the composition of the countries in which, according to the classification of National Health Accounts, a compulsory private insurance scheme is implemented (2). In our proposed framework, this group can be designated as Q-type (Quasi-Market) Social Health Insurance.

The CHI models included in the first and second groups were formed by combining the institutions of the classical Bismarck SHI model and institutions used in the government health financing systems. In this sense, the models of these two groups can be called hybrid, according to their formation and the substitution of institutions of societal regulation by institutions of state regulation. In the original Bismarck model, there were no institutions used in government health financing, while in the CHI models in CEE countries there are such institutions, but the CHI models in CEE countries retain fundamental differences from the government health financing scheme. There are separate systems of financial flows which operate according to the rules set specifically for these systems.

## 6 Conclusion

The paper proposes an approach to analyzing the similarities and differences of country-specific CHI models from the perspective of the regulation of the financial flows in these systems. This made it possible to identify clear similarities between these models, which distinguish them from government health financing systems. The dominance of state regulation in the CHI models in most CEE countries is not a sufficient reason to qualify these models as hybrid financing systems. The general characteristics of SHI are the setting of special rules for the flows of public funds to pay for medical care which are separated from the state budget. There are rules specifying the sources of funds, how they are collected and then allocated among medical service providers. These rules can be set using state, societal, and market regulation in various combinations. The operational management of the movement of funds in SHI can be carried out by different types of financial agents.

These characteristics serve as the basis for distinguishing four types of SHI models: State, Etatist, Bismarckian, and Quasi-Market. CHI models in CEE countries are variations of SHI. Most of CHI models in CEE countries belong to the Etatist, and State types. The only Czech CHI model belongs to the Bismarckian type.

The proposed approach may be used for analysis of CHI models in other countries outside Europe and for international comparison of different national health financing systems. The four types of SHI distinguished may contribute to structuring options when countries make decisions about creation of SHI system or its reforming.

## Data Availability

The original contributions presented in the study are included in the article/supplementary material, further inquiries can be directed to the corresponding author.
